# Environmental sustainability and nutritional quality: addressing global food and nutrition insecurity in a post-pandemic world

**DOI:** 10.3389/fnut.2025.1703994

**Published:** 2025-11-03

**Authors:** Youshen Cao, Yaonan Li, Tianqi Liu, Zixuan Ye, Sangho Lee, Shengkai Geng, Cai Haou

**Affiliations:** ^1^Zhongyuan Wushu Research Institute, Henan University, Kaifeng, China; ^2^Department of Physical Education, Xi'an University of Finance and Economics, Xi'an, China; ^3^School of Martial Arts, Henan University, Zhengzhou, China; ^4^School of Food Engineering and Nutritional Science, Shaanxi Normal University, Xi'an, China; ^5^Department of Physical Education, Northwest University, Xi'an, China; ^6^College of Arts and Sports, Dong-A University, Busan, Republic of Korea; ^7^Institute of Finance and Public Administration, Anhui University of Finance and Economics, Bengbu, China

**Keywords:** food security, nutrition security, climate change, agricultural commodities, policy analysis

## Abstract

**Background:**

This study examines the interrelated issues of food and nutrition insecurity in the post-COVID-19 period, emphasizing the effects of environmental preservation, environmental degradation, and global financial disturbances. Over the last decade, the world has faced unprecedented challenges in food production and delivery, including resource scarcity, biodiversity loss, and substantial price increases. Between 2019 and 2023, over 120 million individuals, representing almost 21% of the global population, experienced food insecurity, underscoring the need to address these challenges.

**Objectives:**

The research utilizes the IMPACT model, augmented with climate projections, agricultural experiments, water cycle evaluations, and global agricultural balance models, to assess the global food supply.

**Method:**

Implies IMPACT model.

**Results:**

The results underscore the need for environmentally sound farming methods and climate resilience to ensure food security in a rapidly changing global landscape.

**Conclusion:**

This study provides thorough qualitative and quantitative perspectives on the intricacies of worldwide nutrition and food systems by examining net accessible calories supply, hunger distribution, micronutrients distribution, and sink calories.

## Graphical Abstract

**Figure:**
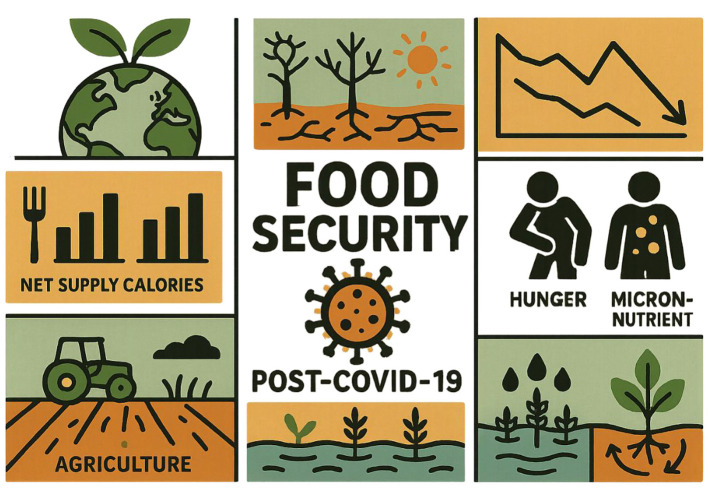
Conceptual illustration summarizing the global food security challenges in the post-COVID-19 era. The graphic depicts interlinked factors such as declining food supplies, agricultural stress, micronutrient deficiency, and hunger risks, with a central emphasis on the “Food Security Post-COVID-19” framework integrating health, nutrition, and agricultural resilience dimensions.

## 1 Introduction

Food and nutrition security has gained significant policy attention; particularly as recent years have revealed a regression in progress. The global count of malnourished populations increased by 21% (122 million) from 2019 to 2023. The factors contributing to this concerning trend include climate change, the COVID-19 pandemic, and escalating global wars, such as the war in Ukraine and civil upheaval in nations like Ethiopia, Myanmar, Sudan, and Afghanistan. The global agrifood system is prejudiced by a confluence of elements, including socioeconomic conditions, dietary shifts, and the overall health of natural resources. The Sustainable Development Goal (SDG 2.1) of eradicating hunger necessitates several years for fulfillment; thus, it is imperative(1). to assess the existing global hunger status and forecast its future trends The world has to deal with these various challenges, many of which pose high levels of uncertainty, to have sustainable food systems to further develop nutrition.

Such obstacles are complicating due to issues like climate change, geopolitical volatility, and the evolving economic environments (2). Foresight practices, both qualitative and quantitative models, have been adopted as a tool to forecast and be prepared in the face of various possible futures. Such models have become more advanced, especially the models that combine factors of biophysical as well as socioeconomic factors to forecast agricultural supply and demand, food security, nutrition, and ecological state (3). Scenario analysis is instrumental in assisting policymakers in analyzing the possible effects of various assumptions and policy options on outcomes like nutrition and population health. The adverse effects of climate change on food security are already being realized, and the nutritional content in raw foodstuffs in most regions has been on the decline. It is especially pronounced in Africa, where climate change has compromised the gains of poverty reduction and economic development initiatives' agenda to enhance food security (4). An evaluation of these indicators will allow us to conclude what role a change in climate has on food insecurity and nutrient shortages. Some areas of interest are found in the study and particularly those whereby the CGIAR network of international research institutions emphasizes to provide relevant information to sway policy making at the national, regional and global levels.

IMPACT was established at IFPRI in early 1991 to address the absence of a strategic plan and agreement among legislators and scholars regarding the measures necessary to ensure global food security, alleviate poverty, and safeguard natural resources. In 1994, these enduring global issues led to the 2021 Plan for Nutrition, Food Security, and the Protection of the Environment Initiative, which ultimately facilitated the development of the IMPACT framework. In 1996, the initial findings utilizing IMPACT were disseminated in an analysis, which examined the influence of individuals, spending, and market situations on food availability and health status, particularly in less developed countries. IMPACT remains the foundation for research investigating the relationship between the manufacturing of essential food products and national consumption and sustainability in potential future scenarios. Research emphasizes geographical concerns, commodity-specific evaluations, and overarching topics. IMPACT is integrated into several significant worldwide assessments to enhance multidisciplinary, scenario-based analyses of the foreseeable future of food supplies and demand. The first comprehensive findings for IMPACT were published in the book Global Food Projections to 2020.

IMPACT modeling method, comprising papers for international organizations containing the United Nations, the World Bank, the Asian Development Bank, the Food and Agriculture Organization, and other national governments. The IMPACT technique has been utilized in regional analyses, including the Asian Economic Disaster and the Prospective World Nutrition Conditions, both of which were authored in reaction to the 1997–1998 Asian financial crisis and aim to evaluate its effects on the surrounding agricultural economic system. examined escalating nutrition challenges in the Arab area. A prominent discussion paper from the IMPACT team was their presentation to the 2030 Agenda summit in Uganda on “Ensuring the Security of Nutrition and Food in the African continent by 2022.” Instances of commodity-centric research are evident in the study examining the correlation between meat-heavy diets in prosperous nations and food availability in developing nations, as seen in Alternatives Prospects for International Agriculture and Food Usage.

## 2 Methodology

**Figure 1:**
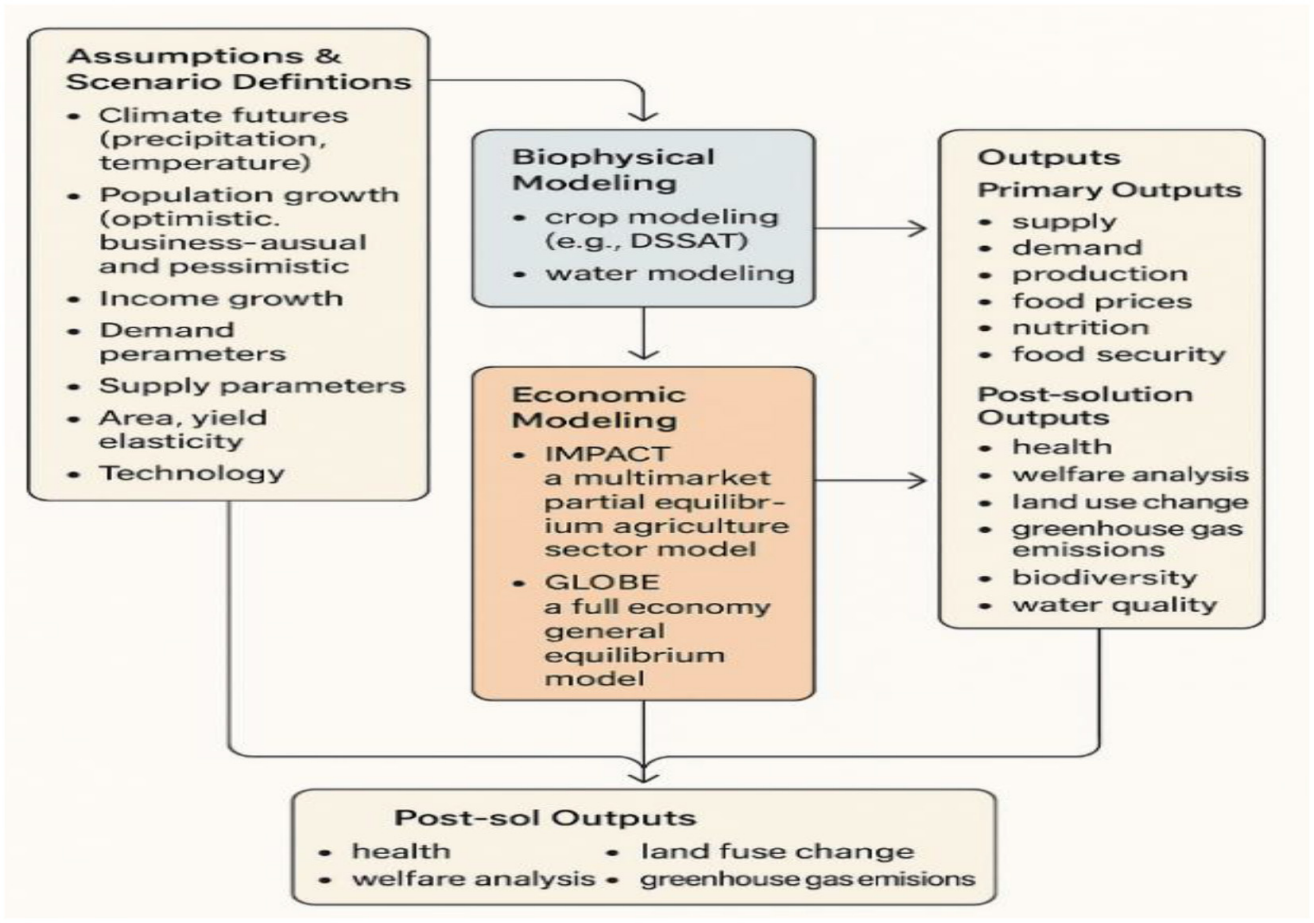
Framework for extended IMPACT modeling.

The overall structure of the extended IMPACT modelling framework is presented in Figure 1, which outlines the flow from assumptions and scenario definitions through biophysical and economic modelling to final outcome variables.

### 2.1 IMPACT model and updates for food security forecasting

The IFPRI has an IMPACT network, where crop simulation models (such as DSSAT to Agro technology Transfer), climate models, and water models fit into the network, which uses a mass of combined models that are put together, to digest many data sources. Global, partial equilibrium, and based on the agricultural sector is its fundamental paradigm. Mason-D'Croz et al. (5) claim that this model of the economy depicts the production, demand, and trade by 158 countries in 62 different forms of agricultural goods. Researchers and policymakers can use the IMPACT system as a whole to compare and analyze biophysical systems, socioeconomic changes, technology advancements, and policy proposals. Under the IMPACT paradigm, the main components of the undercarriage scenarios are income (as measured by GDP), population increase, and assumptions regarding hypothetical important agricultural productivity growth (6). The data on the GDP and the growth rate of population in this model are obtained in the Shared Socioeconomic Pathways (SSP) repository by the International Institute for Applied Systems Analysis under the Intergovernmental Panel on Climate Change. These data are essential when forecasting future patterns regarding food security, and can be used to analyze a specific situation on a long-term level. Two important updates have been carried out to make the model more accurate. First, the model base year has been changed to a 3-year average of the latest FAOSTAT data, with the average year of 2019 (7). Second, the rates of increased crop yield growth have been re-estimated by Bayesian inference. This procedure is based on the use of previous yield estimates (the priors) and additional information, namely the latest crop yield statistics of FAOSTAT (8). These yield growth rate assumptions were first formulated in previous research that reviewed the trends in yield growth that occurred as a result of farming research, market infrastructure, and extension programmers, with data provided by FAOSTAT. These projections have been modified over time by expert consultations, model comparisons, and updates to agricultural research expenditures (9). The model further combines the recent growth in yields from 2009 to 2018 with previous long-term yield growth forecasts, where weighted averages are used to generate yield growth assumptions that are more credible in the future. The weights of the 2019–2024 period are 0.4 (the last FAOSTAT regression yield growth rates) and 0.6 (the last long-term growth rates). Such a combination of the past and the present forecasts will provide a balanced and accurate evaluation of future trends of agricultural productivity (10). Such updates will help the IMPACT framework to provide a more refined approach to the issue of food security against a shifting climate and socioeconomic context.

The FAO regression weight for yield increase decreases at a rate of 0.1 per 5 years, reaching a value of 0.0 between 2040 and 2050. Thus, short-term trends are paramount in the years immediately following 2020, with their influence gradually diminishing thereafter (11). The model is built based on future climate scenarios based on the present bias-adjusted ISIMIP3b database (12) that has been downscaled to CMIP6 climate scenarios (13). The IPSL-CM6A-LR general circulation model (GCM) was used in this analysis, and sensitivity testing is being run currently to compare the performance of this model with that of other general circulation models. Climate change scenarios used follow two basic elements. The Shared Socioeconomic Pathways (SSPs) represent the initial framework and indicate alternative trajectories for economic and demographic development (14). The current study utilizes SSP2 as the reference scenario, characterized by moderate GDP and population growth. The second element is Representative Concentration Pathways (RCPs), describing the potential future concentrations of greenhouse gas emissions and the resulting increase in the amount of solar energy absorbed (15). We apply RCP7.0, the more favorable climate change scenario, but not so harsh as RCP8.5. It is currently viewed as very unlikely, but is used as an upper-bound scenario. In the year 2050, the difference between growth in climate scenarios is not very significant, but in the following years, it grows tremendously. In our calculation, we take into account the factors of climate change, temperature, and precipitation on crop yield. Fertilization by CO_2_, in the reference (not a case study), but possibly enhancing the yield, should not have any effect (16). However, the long-term consequences of CO_2_ fertilization would differ among crops, sites, endowment of resources, and management, and studies are underway on the consequences. The sensitivity analysis is conducted to approximate the potential influence of CO_2_ fertilization on food security.

### 2.2 Per capita food demand and nutrient supply in the IMPACT model

The projected per capita food demand of the average consumer in every region is one of the primary outputs of the IMPACT model. Each commodity is determined as the per capita kilocalorie and nutrient supplies using region-specific technical coefficients, and the data is grouped into commodity groups per geographic unit after accounting for consumption patterns.

This approach facilitates the examination of the correlation between water supply and food demand across many geographical scales, including watersheds, nations, more integrated zones, and the world level. Water availability and demand, along with crop output, are first evaluated at the watershed level. Subsequently, agricultural output is aggregated to the national level, where food consumption and commerce are simulated. The previous IMPACT model categorized the entire globe into 40 countries and territories; however, it was subsequently refined into 300 “food-production units.” These FPUs indicate the geographical convergence of 114 socioeconomic (predominantly geographical) areas and 125 waterways basins, acknowledging that considerable climatic and hydrological variability within geographical areas render the utilization of large geographic areas unsuitable for water supply evaluation and modeling purposes. Among the nations included in the IMPACT-WATER model, the Asian nation of China and the USA, which collectively account for approximately 65% of global agricultural output, exhibit the greatest degree of territorial segmentation, being partitioned into 8, 15, and 17 principal river basins, respectively. In contrast, the other countries or regions analyzed in IMPACT are aggregated into the remaining 95 watersheds.

#### 2.2.1 Model for estimating future hunger risk

The model assumes the number of people at risk of starvation in the future by estimating the percentage of the population with a minimum amount of calories eaten below a specific level of minimum caloric consumption. It is analogous to the FAO measure of undernourishment, which is a leading indicator of food insecurity (17). The approach uses the SSP2-based projections that assume an increase in population. The approaching empirical formula underlying the methodology is the strong relation between per capita availability of food and the level of the number of people who are exposed to hunger. In particular, it assumes that the occurrence of the caloric intake about the mean of its per capita caloric availability is normal. The model calculates the fraction of the population that falls below the minimum caloric requirement by incorporating the density function up to the extent needed to attain the caloric requirement (18). This gives an approximation of the population that is in danger of starvation in various areas. Findings of this model are essential in evaluating the possible effects of several socioeconomic and climate change scenarios on world hunger.

#### 2.2.2 Micronutrient availability

Another major measure that the IMPACT model uses is the availability of micronutrients in regional diets. The population-level availability of key micronutrients (iron, zinc, and vitamins) based on the average regional diets modeled by Beach et al. (19) is calculated using methods developed. These estimations are made to consider the normal food preparation habits in a particular country. After the availability of micronutrients is known, the model contrasts this with the recommended nutrient intakes (RNIs) in existing literature, which differ depending on the specific population characteristics in each country (20). The most important measure is the RNI ratio, meaning the ratio of available nutrients to recommended intakes. A ratio of RNI 1 shows that the average nutrient intake in the population is satisfactory. Conversely, a value below 1 indicates deficiency, and a ratio above 1 indicates excess supply of nutrients. These ratios provide useful information on the sufficiency of diets at the population level, and they indicate a possible imbalance of micronutrients or an overload of nutrients that could lead to larger health problems (21). It is important to note, though, that these RNI ratios are population-based measures, and distributional concerns at the country level can result in disparities in nutrient access. Certain groups of people can experience a deficiency of nutrients, whereas some groups can experience sufficient or excessive availability of food rich in micronutrients.

#### 2.2.3 CGIAR focus areas in IMPACT model

The IMPACT model is internationally applicable and mimics the agricultural systems of 158 nations and 62 commodities. However, to enhance efficiency and clarity in presenting data, specific aggregations are conducted. Results are presented with a focus on six primary areas of particular interest to the CGIAR network of worldwide research institutions (22). This summary delineates the six areas of interest to the CGIAR. These aggregations streamline the voluminous data generated by the model, enhancing its accessibility and utility for regional and global policymakers.

### 2.3 GDP and population growth projection under SSP2 scenario

**Figure 2:**
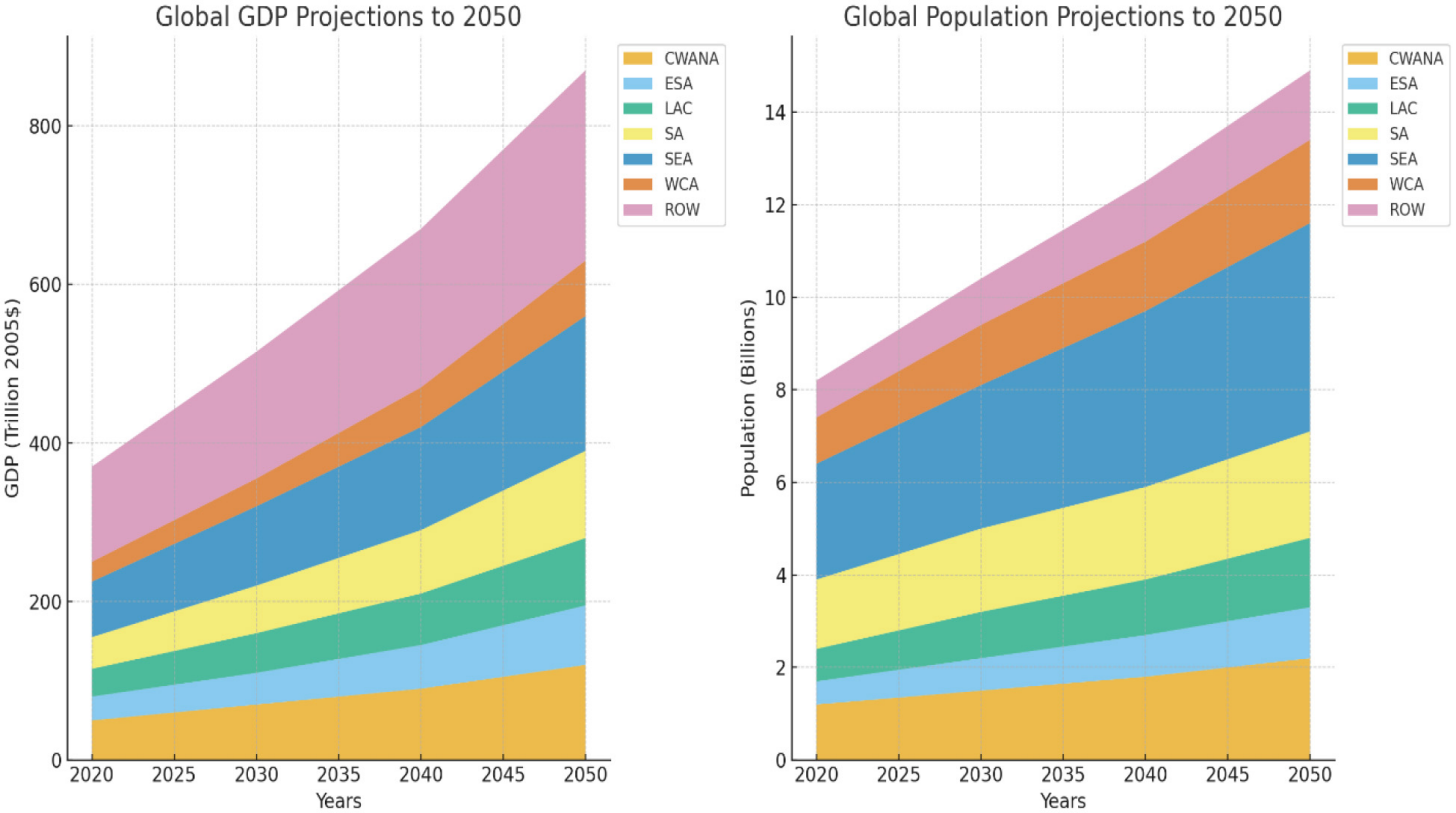
Global GDP and population growth through the middle of the century, using the second shared socioeconomic pathway.

The projections of growth of GDP and population by region under the SSP2 scenario. The SSP2 can be considered a moderate scenario, and assumptions about the future modernization of the economy and population are moderate. Here, the increase in the world GDP is projected to exceed threefold in the years 2020 to 2050, which will increase the figure to 101 trillion to 231 trillion. The GDP growth rate of developing countries is forecasted to be 3.53% per annum compared to only 1.62% per annum in the developed countries. The highest growth rates are in Africa, with East and Southern Africa (ESA) expected to increase by 4.83% per annum and West and Central Africa (WCA) at 5.78% per annum. Nevertheless, the regions have a relatively low starting GDP. The projected GDP and population trends for different world regions under the SSP2 scenario are shown in Figure 2, indicating consistent economic expansion and demographic growth toward mid-century.

## 3 Results

The simulation exercise of the world market production, demand, and world prices is the IMPACT model, which examines the national and international market dynamics in order to balance the supply and demand in the world market. The model balances the equilibrium price and clears the global markets, that is, supply, crop production, and animal production become the same as the demand, and trade all commodities. Here we discuss the findings of the reference scenario, i.e., the food and the projection of the kilocalorie requirements, the projections of the yield and the production area, and the projections of the prices of the commodity.

### 3.1 Food and kilocalorie demand

**Figure 3:**
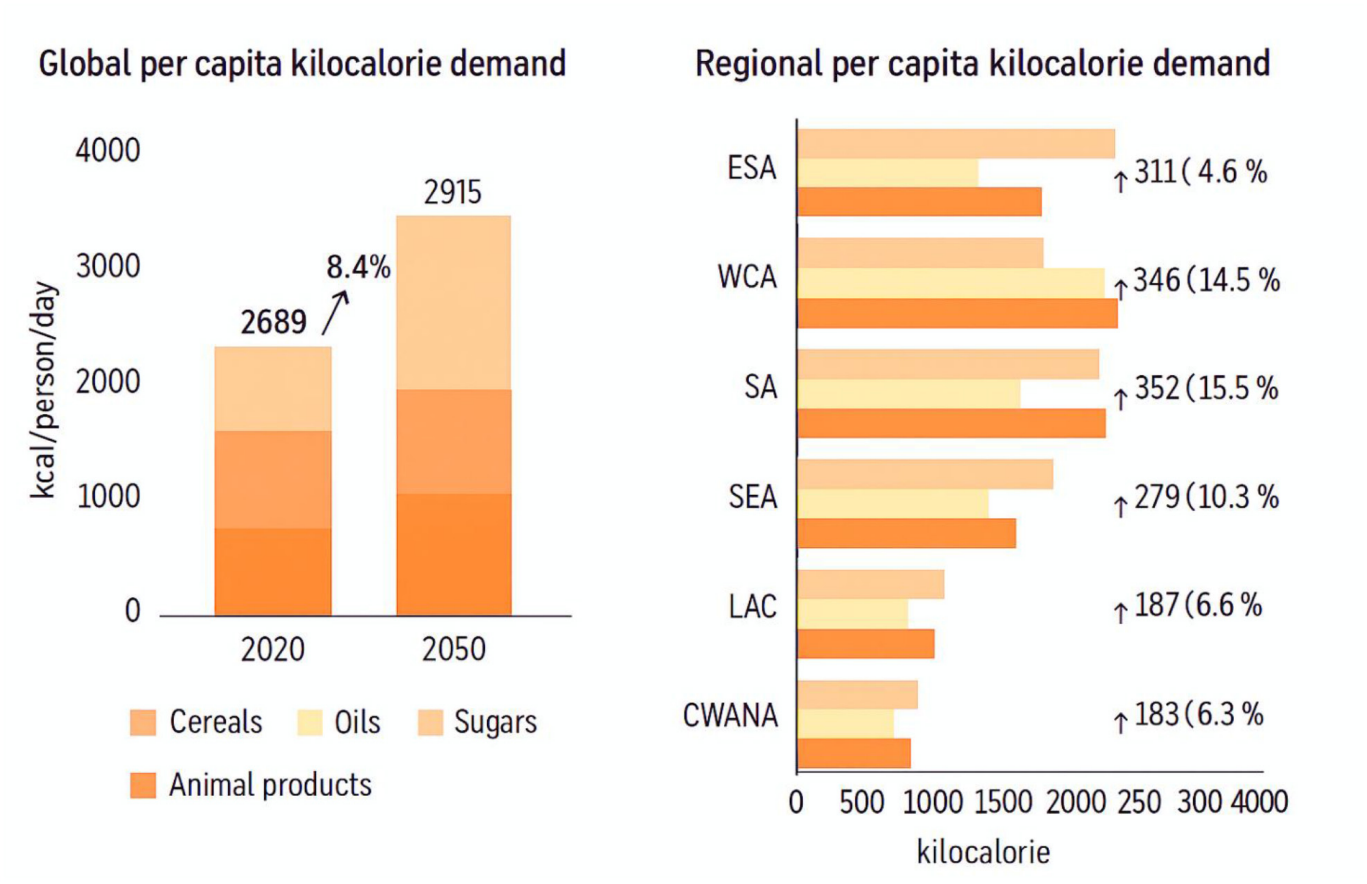
Global per capita kilocalorie demand and regional per capita kilocalorie demand.

The simulated global and regional changes in per-capita kilocalorie demand are illustrated in Figure 3, highlighting regional dietary transitions and rising calorie requirements across developing economies. The worldwide per capita kilocalorie demand is projected to increase by 10% from 2018 to 2060, ranging from 2,789 to 2,919 kcal/person/day. Countries that are developing are predicted to experience the most significant surge in kilocalorie consumption, with an expected growth of 11% to 2,820 kcal/person/day by 2055. In contrast, industrialized countries would see a modest gain of 2.973%, reaching 3,351 kcal per person per day by 2060. The main suppliers of calories are grains, oils, sugars, and animal products, which provide the fundamental base of per capita food now and in future generations. By 2020, these products are expected to account for 46.8%, 19.7%, and 17.9% of the global average nutrition, respectively. By 2020, the typical global diet is anticipated to undergo modifications. The share of grains in the global diet would decrease by three percentage points, while the amount of oils and sugars would increase to between 19.34 and 20.976%. The proportion of agricultural products is expected to rise by a modest 1.5%. Dietary expansion is more pronounced in emerging countries, where the proportion of cereals is projected to decrease by 4%−5% points.

In contrast, the proportions of other food categories are expected to rise by less substantial ranges. The demand in emerging nations is expected to increase significantly in total kilocalories: grains are projected to rise by 3%, livestock products by 25%, food items by 28%, and oils and sweets by 27%. The anticipated variations in kilocalorie requirement are also categorized by area. The greatest rise in per capita kilocalorie accessibility, both in absolute terms and percentage, is anticipated in East and Southern Africa (ESA), with a rise of 312 kcal/person/day (15.7%).

### 3.2 Projected agricultural changes 2020–2050

The expected changes in production, yields and land use will vary based on shifting demand, advancements in technology and impacts of climate change. Table 1 demonstrates the estimated changes in these variables between 2020 and 2050 in the different regions. These changes are internal to the model, i.e., they occur as a result of interactions within the agricultural system. The growth in reference yields, which is explained in the methodology section, adapts to market equilibrium and global prices (23). It is projected that animal product production is going to increase by 43% in the world, 55% in the developing world, and 22% in the developed world (Table 1a). Global grain output is expected to increase by 40%, with the developing world experiencing a 49% rise and the developed world a more moderate 20% growth. This imbalance is driven by rapid population growth, urbanization, and the modernization of farming in emerging areas, characterized by abundant undeveloped land and opportunities for increased production. In contrast, industrialized countries face land limitations and a transition toward a broader variety of agriculture.

Table 1Projected percentage variation in production, yields, and harvested area for major commodity groups from 2020 to 2050 according to the IMPACT model.

**Table:** 

**Region**	**Animal products**	**Cereals**	**Oils and sugars**	**Roots and tubers**	**Fruits and vegetables**	**Pulses**	**Others**
**(a) Production**							
CWANA	73.6%	36.7%	59.9%	44.0%	71.2%	39.9%	64.7%
ESA	81.3%	76.0%	65.6%	89.1%	101.3%	39.9%	137.3%
LAC	41.5%	87.0%	52.8%	40.3%	42.2%	60.0%	53.3%
SA	81.3%	48.4%	29.4%	67.0%	114.8%	21.9%	−22.7%
SEA	39.4%	14.3%	56.9%	1.3%	25.3%	41.3%	21.9%
WCA	123.2%	56.5%	78.5%	49.9%	76.2%	121.8%	54.8%
ROW	15.9%	34.5%	44.6%	6.1%	23.5%	53.5%	48.2%
DVD	21.8%	19.2%	41.5%	13.5%	36.5%	49.2%	50.8%
DVG	55.3%	46.1%	51.5%	33.7%	49.3%	52.7%	28.6%
WLD	43.3%	37.8%	50.0%	31.8%	47.8%	51.7%	30.9%

**Table:** 

**Region**	**Cereals**	**Oils and sugars**	**Roots and tubers**	**Fruits and vegetables**	**Pulses**	**Others**
**(b) Yields**						
CWANA	28.6%	32.9%	18.3%	34.7%	26.8%	43.6%
ESA	41.1%	30.5%	58.2%	34.2%	37.7%	87.2%
LAC	35.2%	29.1%	22.6%	19.9%	38.7%	29.9%
SA	38.3%	13.7%	16.6%	45.6%	46.7%	11.1%
SEA	12.2%	17.8%	6.8%	34.3%	11.4%	1.1%
WCA	25.6%	16.8%	21.7%	32.1%	22.1%	24.0%
ROW	31.1%	46.6%	12.4%	21.7%	41.5%	20.4%
DVD	22.4%	37.0%	13.9%	22.5%	37.9%	21.5%
DVG	29.8%	24.4%	15.5%	22.7%	42.4%	22.0%
WLD	25.7%	27.7%	14.4%	22.5%	41.5%	22.2%
**Region**	**Cereals**	**Oils and sugars**	**Roots and tubers**	**Fruits and vegetables**	**Pulses**	**Others**
**(c) Areas**						
CWANA	6.3%	23.0%	21.7%	27.1%	10.3%	14.7%
ESA	24.8%	28.4%	19.5%	50.0%	35.8%	26.7%
LAC	38.3%	19.2%	14.3%	35.9%	15.4%	18.1%
SA	7.6%	13.6%	43.5%	9.9%	−16.9%	−23.5%
SEA	1.8%	−1.5%	−5.1%	11.0%	5.2%	9.4%
WCA	24.6%	46.1%	23.1%	33.3%	37.1%	24.8%
ROW	−2.6%	0.4%	9.9%	5.1%	8.2%	23.3%
DVD	−6.2%	4.3%	2.3%	21.6%	7.4%	20.4%
DVG	9.8%	21.0%	15.5%	22.7%	42.4%	22.0%
WLD	9.6%	17.4%	15.2%	22.5%	7.2%	7.1%

Global cereal yields are projected to rise by 28%, with poor countries anticipating a 34% increase, in contrast to a 25% rise in affluent nations. Developing areas, characterized by lower production, would benefit from enhanced agricultural techniques, superior seeds, and improved transportation. Developed nations, with higher production levels, will continue to see incremental advancements through technical developments. The global grain manufacturing region is expected to increase by 10%, with established countries experiencing a 4% decline and emerging regions expanding by 16%. This transition signifies the increased availability of cultivable land and the growing demand in emerging countries.

The developing world is expected to experience a significant increase in grain output, yields, and acreage, driven by enhanced capacity for improvement and rising food demand, while industrialized nations will encounter slower development due to land constraints and environmental regulations. East and Southern Africa (ESA) is projected to experience the most significant rise in output, albeit from a low baseline, offering considerable potential for improvement.

Meanwhile, Southeast Asia (SEA) is expected to have the least yield increases, especially with cereals, as initial yields are high, and input is used in 2020. It is estimated that strong yield growth in cereals, fruits, vegetables, and pulses will be achieved in South Asia (SA). The increase in production acreage, however, varies much more between developing and developed nations, with the increase in area being higher than the increase in yield, especially in the case of pulses and other commodities (24). The potential increase in agricultural areas is low in developed countries, and in ESA and WCA, most crops will see the same growth in area. Latin America and the Caribbean (LAC) is projected to experience the most substantial growth in crop area, particularly with an anticipated increase in oils and sugars. Conversely, a decline in the crop area for cereals, roots, and tubers is expected in Southeast Asia (SEA). The decline in production of alternative crops in developing countries, particularly in South Asia, can be primarily attributed to the reduction in the land area allocated to these crops. Area, yield, and production trends are projected and analyzed in comparison to historical trends from 2000 to 2020; the estimates align with the developments observed over the years, albeit at a reduced rate.

### 3.3 World food prices

**Figure 4:**
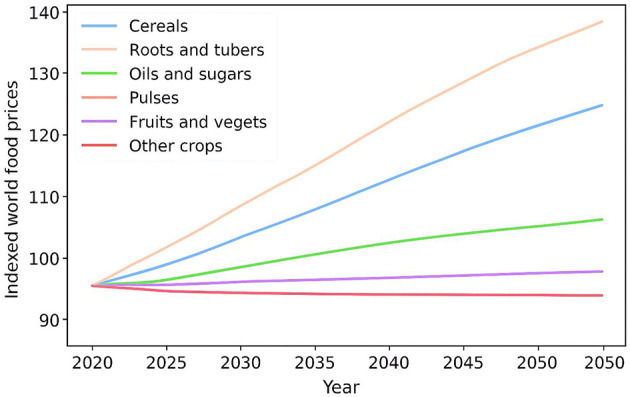
World food prices.

The IMPACT model, as mentioned above, reflects the dynamics of the national and international market, as production, demand, and global prices are modified until a balance is reached between supply and demand on the global scale. As a result, world food prices are endogenous to the model and vary according to these market forces. The projected weighted world prices of the various commodity groups in the SSP2 and RCP7.0 indexed in 2020. According to the model, the world prices of most of the commodity groups are expected to increase by 2050, except that the pulses are expected to remain constant over the period. By the year 2050, the prices of cereals, fruits, and vegetables, and other crops are likely to increase by more than 20%. It is believed that oils and sugars will record a more modest price rise of 7%. In the meantime, the price of animal products will be at the highest point in the year 2035 before starting to decrease. The greatest price increase is expected in roots and tubers, which are likely to experience a 37% improvement over 2020. Among the significant contributors to the rise in cereal prices are the projected rise in demand for maize, particularly in livestock food and biofuel like ethanol. U.S. and China will contribute more than a third of the global maize demand, where the growth in demand is largely driven by consumption of feed and ethanol (25). In China, feed demand contributed to 71% of maize demand in 2019, and in 2050, feed demand will increase more than twice, and feed will take up more than 83% of the total. Likewise, in the U.S., almost half of the total maize consumption was used in ethanol production in 2022, with the livestock feed occupying 40%. By the year 2050, it is estimated that the U.S. will experience a 50-fold rise in the demand for maize. Even though ethanol and feed shares are expected to fall by only a few points, they will continue to be the leading drivers of the increase in maize demand (26). In 2050, half of the maize will be consumed by ethanol and 28% by livestock feed. Food demand is majorly increased by population growth and increasing incomes. With the growing demand, production will also increase, but at a rising cost because of the limitations of resources and technology. Although the model forecasts long-term patterns in food prices, short-term price shocks and variability are not represented in the model (27). Crises like the COVID-19 pandemic or the Russia-Ukraine conflict can result in a substantial change in food prices, which in turn may cause a short-term price rise that is even higher than projections given by the model in the long term. The projected evolution of world food prices for key commodity groups is shown in Figure 4, demonstrating upward trends in cereals, roots, and tubers under the SSP2-RCP7.0 scenario.

### 3.4 Global hunger projections and SDG 2.1 progress

By 2020 to 2050, the global community is likely to achieve some progress in decreasing hunger. Nevertheless, this development is expected to be gradual and not enough to meet global targets to end hunger. The Sustainable Development Goal 2.1 (SDG 2.1) of the United Nations is to eliminate hunger by 2030, and the target is the reduction of hunger to 5% of the world population (28). This goal is informed by the 2015 report by the FAO on Achieving Zero Hunger, which uses a 5% decline in the population to determine the end of hunger. It is projected that developing nations will eliminate 661 million hungry individuals in 2020, 583 million by 2030, and 453 million by 2050. In 2020, South Asia accounted for 38% of the global population at risk of hunger and is anticipated to achieve the most significant reduction in hunger levels. By 2030 and 2050, hunger in South Africa is expected to be reduced by 56 million and 131 million, respectively. Improvements are anticipated as a result of projected increases in income and technological advancements; however, addressing the impacts of climate change remains complex. Conversely, the projections show thwarted progress in hunger alleviation in East and Southern Africa (ESA) and the West and Central Africa (WCA) (29). Population growth in these areas may outpace improvements in food security, leading to negligible changes in the proportion of individuals at risk. Similarly, population growth in Central and West Asia (CWANA) is anticipated to result in a slight growth in the number of individuals at risk of starvation.

Southeast Asia (SEA) has achieved a lot in alleviating hunger over the past decades and is likely to keep gaining momentum, albeit with a slower pace. The reductions in hunger will also be recorded in Latin America and the Caribbean (LAC). However, these regions will not achieve the SDG 2.1 target of halving hunger to 5% of the population by 2030 (30). Presents the anticipated share of populations at risk of being hungry by region. Most regions and developing countries will hardly achieve the SDG 2.1 target by 2030, despite the progress made. The goal has already been achieved, but SEA will still improve, and in 2020, only 3% of its population will be at risk of starvation. By 2040, LAC will exceed the target of 5%. South Asia, with significant growth, will only achieve the 5% target by 2050, which was 14% in 2020.

**Figure 5:**
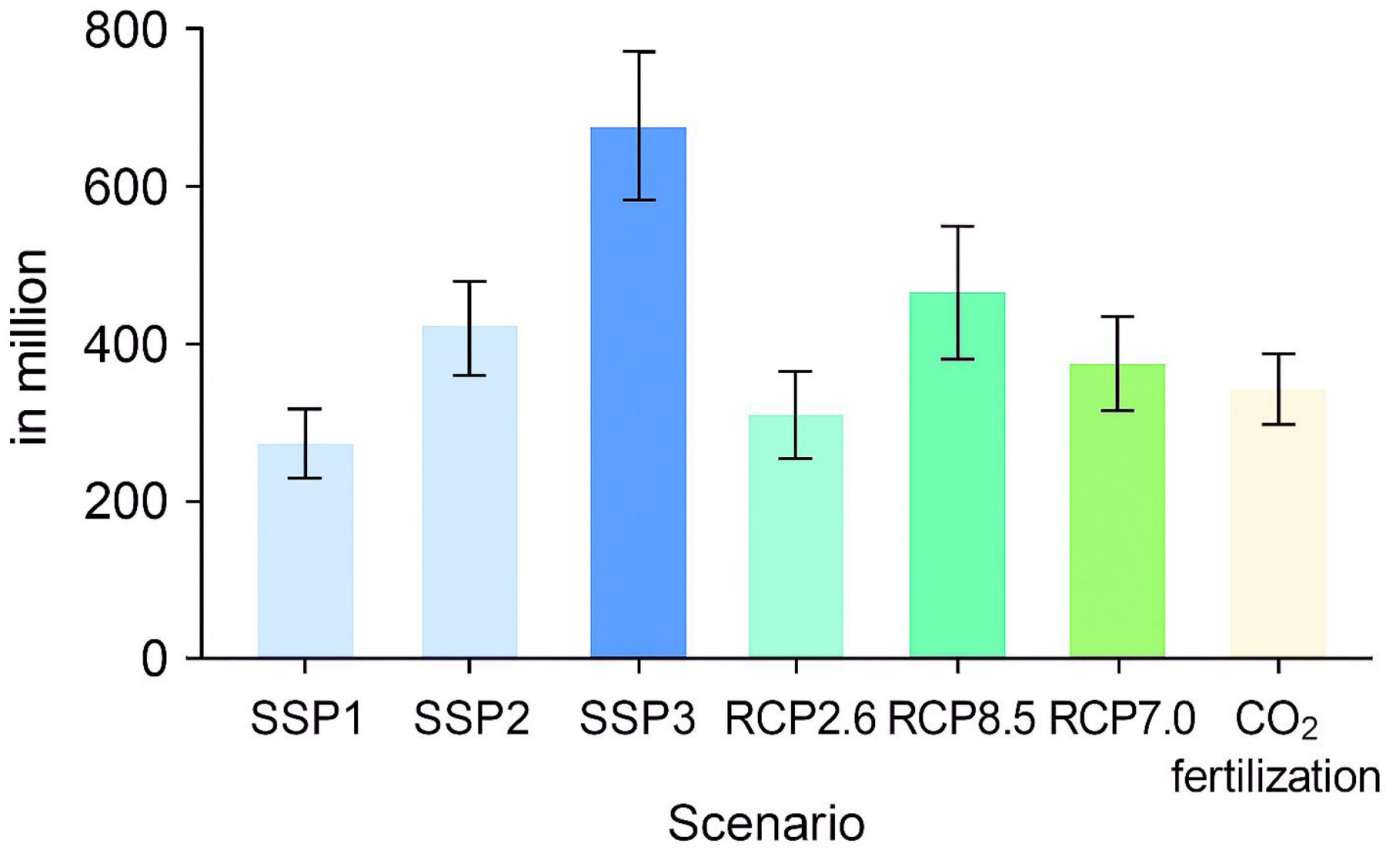
Number of individuals at risk hunger.

In comparison, both ESA and WCA are likely to have 24 and 14% of their populations at risk of hunger in 2030, respectively, and fail to achieve the 5% target by 2050 because the levels of hunger are high in 2020. By 2030, CWANA is projected to have 7% of its population at risk of hunger, and the decline will be slow thereafter. The proportion of individuals vulnerable to hunger is expected to decline to 8% in 2030 and 6% in 2050, in all developing countries, making some consistent but inadequate progress toward achieving the global hunger target. The estimated number of individuals at risk of hunger under the reference and alternative scenarios is summarised in Figure 5, illustrating regional disparities and the persistence of food insecurity beyond 2030.

### 3.5 Micronutrient availability

**Figure 6:**
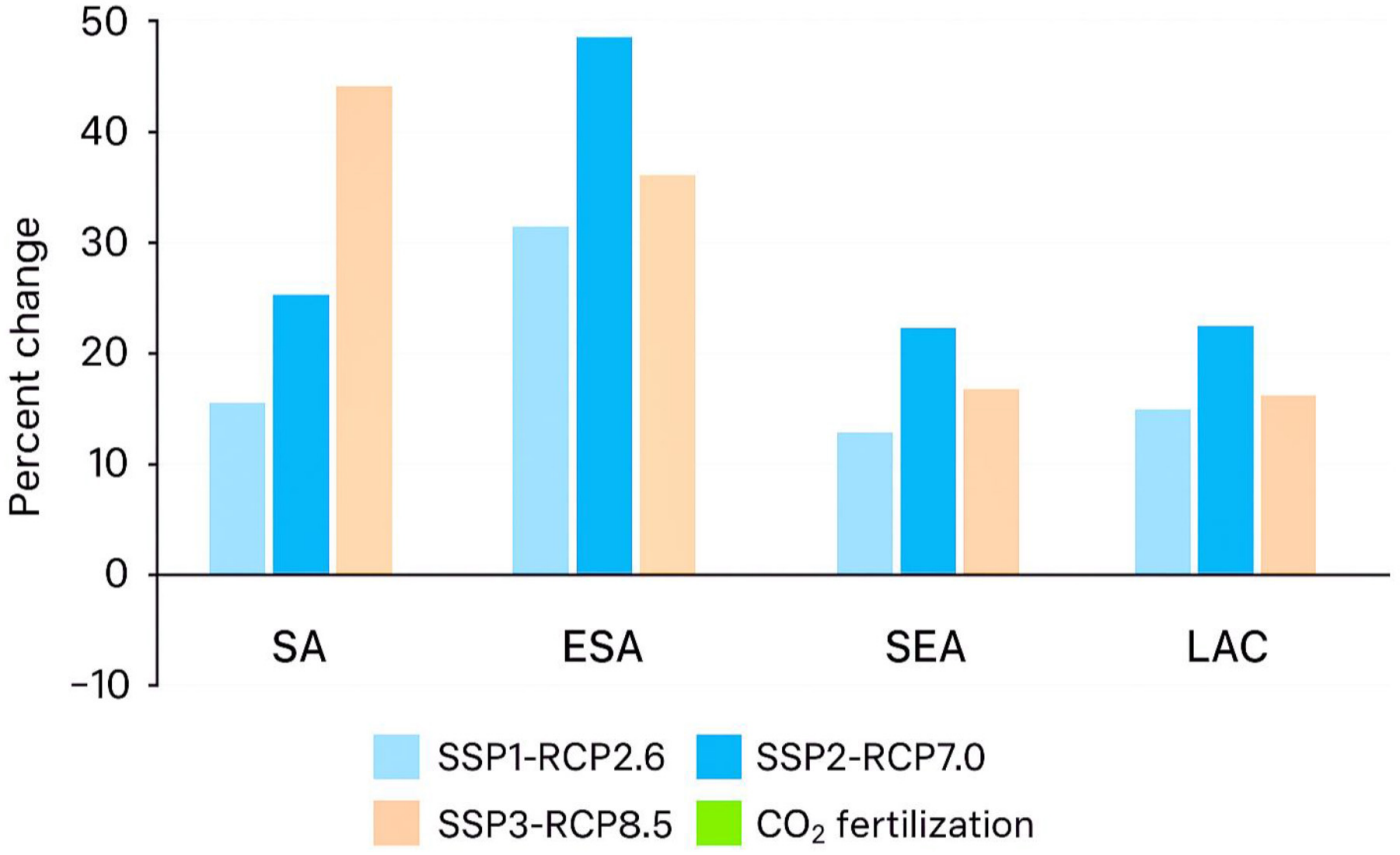
Changes in nutrients availability in diets.

In addition to the estimated overall growth in world food supply, the supply of micronutrients in diets is also increasing. Areas like South Asia (SA), East and Southern Africa (ESA), and West and Central Africa (WCA), which initially have relatively low levels of micronutrient availability in 2020, will experience considerable growth in per capita nutrient availability (31). Nutrient availability in SA will rise by 46% between 2020 and 2050, and ESA will see a similar rise of approximately 50%, with other regions recording smaller gains. Overall, alternative scenarios simulated in this analysis would generally result in a nutrient availability difference of/- 10% compared to the reference case in 2050 (SSP2-RCP7.0). The ratios of the recommended nutrient intake of the major nutrients, namely, protein, iron, and zinc, indicate the changes in nutrient availability as compared to the recommended levels of nutrient intake (Table 2). The availability of protein is typically adequate throughout the regions, and the ratios of RNI are always greater than 2. Regional variations and projected changes in nutrient availability across baseline and future scenarios are depicted in Figure 6.

**Table 2:** Typical regional protein, iron, and zinc availability-to-recommended-nutrient intake ratios (“RNI ratios”).

**Region**	**Protein (2020)**	**Protein (2035)**	**Protein (2050)**	**Iron (2020)**	**Iron (2035)**	**Iron (2050)**	**Zinc (2020)**	**Zinc (2035)**	**Zinc (2050)**
CWANA	2.70	2.90	2.86	1.31	1.41	1.67	1.72	1.98	1.90
ESA	2.40	2.76	2.87	0.78	1.20	1.41	0.78	1.17	1.31
LAC	2.50	2.63	2.86	1.41	1.55	1.43	2.78	2.90	2.45
SA	2.31	2.70	2.97	0.44	0.71	0.80	0.88	0.67	1.89
SEA	3.38	3.70	3.75	1.91	1.90	2.79	2.62	2.90	2.94
WCA	2.32	2.51	2.80	0.98	1.11	1.71	1.90	1.22	1.31
DVD	2.80	2.90	2.99	1.90	1.91	1.83	2.88	3.45	3.22
DVG	2.60	2.85	3.54	1.02	1.15	1.31	1.67	1.78	1.98

Nevertheless, iron and zinc deficiencies remain common in a few regions. Southeast Asia (SEA) and Latin America and the Caribbean (LAC) will probably enjoy more dietary iron and zinc than South Asia and ESA (32). As mentioned above, the distribution issues will impact the access to healthy diets, and people belonging to the lower-income group will experience more severe shortages than the average regional values indicate. The RNI ratios in other situations suggest that positive drivers, including changes in socioeconomic conditions, can be used to achieve improved nutritional results. A more difficult socioeconomic environment, on the contrary, can slow progress. Globally, cereals are the main source of protein, iron, and zinc (and other nutrients), and the second contributor is animal-based foods. Also, depending on the region, pulses, fruits, and vegetables are important to boost RNI ratios.

### 3.6 Sensitivity analysis

Various assumptions are sensitive to the results of hunger outcomes, including assumptions about economic and population growth, scenarios of climate change, CO_2_ fertilization, and application of various General Circulation Models (33). These assumptions are tested with the comparison of alternative conditions: economic growth forecasts with SSP1, SSP3, and the seed forecast of the reference SSP2, climate change scenarios (RCP2.6, RCP8.5, and RCP7.0), the inclusion of CO_2_ fertilization in crop yield forecasts, and the use of the output of five different GCMs (including the reference IPSL model and GFDL-ESM4, MPI-ESM1-2HR, MRI-ESM2-0, and UKESM1-0-LL). In the example above, the population of the world is estimated to reach 9.2 billion in 2050 in the reference scenario (SSP2), with the world GDP amounting to 231 trillion. However, the other socioeconomic scenarios, SSP1 and SSP3, offer different perspectives. The population will be projected to grow to 8.6 billion under SSP 1, and the global GDP under SSP 1 will be much higher at 287.4 trillion, showing a more positive economic growth pattern.

On the other hand, SSP3 also considers a less optimistic future, having a world population abundance of 9.9 billion and a global GDP estimated at 179.1 trillion. These disparities in economic and population assumptions lead to different levels of hunger outcomes, with SSP1 recording the biggest reduction in hunger and SSP3 recording a significant increase in the number of people who are going to be hungry (34). The other climate scenarios (RCPs) also have an impact on hunger outcomes, where RCP2.6 indicates a lower climatic change, RCP7.0 is the reference, and RCP8.5 indicates the highest magnitude of climatic change. To illustrate, the world population of hungry individuals is projected as 375 million under RCP2.6 (4.1% of the population at risk) compared to 466 million under RCP8.5 (5% at risk). The climate scenarios also influence the outcomes of hunger, but are outweighed by the impacts of the socioeconomic conditions.

The CO_2_ fertilization is another variable in the sensitivity analysis and can translate into a higher yield of crops through enhanced photosynthesis. There is uncertainty regarding the effect of CO_2_ fertilization on hunger, as some studies have shown that it can cause positive yield growth, particularly in some crops under certain conditions (35). To investigate this, the model plays out a scenario in which CO_2_ fertilization is added to the crop yield projections. The number of individuals around the world who would be vulnerable to hunger in the year 2050 in this scenario will be 382 million, as opposed to the 481 million in the reference condition, where CO_2_ is released into the soils. This ratio of the population at risk is also narrowed to 4.2%. These findings suggest that CO_2_ fertilization may have some alleviation benefits in reducing hunger, but its efficiency remains questionable and region-dependent and crop-dependent (36). The difference in the output between the various GCMs is relatively minor, which are the upper and lower projections of every scenario. This implies that GCMs are offering more varied forecasts about climate change, yet, on the whole, they have no drastically different effects on hunger outcomes. Nonetheless, regional outcomes, presented in additional demonstrate that certain regions are susceptible to climate change scenarios than others. Specifically, the areas where hunger is very vulnerable to climate change are West and Central Africa (WCA), South Asia (SA), and East and Southern Africa (ESA). The number of hungry people in these regions is also expected to vary more significantly depending on the magnitude of climate change (37).

In comparison, SSP1 would reduce the number of hungry people by 43% by 2050 to a total of 257 million hungry people, whereas in the reference scenario (SSP2), there would be 453 million hungry people. However, in the case of SSP3, the increase is drastic, as the population of hungry people is estimated to grow to 780 million, 72% more than in the reference scenario and 116 million more than in 2020. The risk of hunger will amount to 3%, 5%, and 8% of the population in 2050 according to SSP1, the reference scenario (SSP2), and SSP3, respectively. This indicates that the most important factor in the determination of hunger outcomes is socioeconomic assumptions, and optimistic assumptions would cause a significant decrease in hunger, and pessimistic assumptions would cause a significant increase.

The number of individuals at risk of hunger depending on the different scenarios, where the bars indicate different assumptions made about SSPs, RCPs, and CO_2_ fertilization. The whiskers show the highest and lowest values of each of the GCMs in each scenario. The percentage of the population under the risk of hunger in the same set of conditions, and it is quite easy to see that there is a considerable variation in the assumptions made in SSP1, SSP2, and SSP3. There is also a similarity between the global results and the regional results of the scenarios. The highly sensitive areas to the alternative climate scenarios would be WCA, SA, and ESA.

### 3.7 Limitations of the study

The limitations of this study are related to the global long-term modeling. Although the Shared Socioeconomic Pathways (SSPs) offer useful forecasts on economic and population growth patterns, other important factors driving global food security are not considered by the projections. As an illustration, in this analysis, the main changes with climate change are long-term variation in temperature and precipitation (38). Nevertheless, it does not consider any other factors like climate change, extreme events, sea level rise, or glacier melt, which can influence some areas. Moreover, poor governance, unstable states, and war can deter economic and productivity development, which is not the case in the reference scenario. The model fails to consider the effects of unexpected shocks, like the COVID-19 pandemic, which may change the course of food security in the short term. The IMPACT model posits that agricultural land may expand in response to fluctuations in commodity prices. It fails to address the competition between land use and agriculture, which can erode land availability for land use later, particularly in those locations that already have other land use purposes. The land intensification is also not considered in the model and might be needed in the case of land scarcity. These could influence the accuracy of the projections, especially where the land area is small.

## 4 Conclusion

The paper presents the projections of food and nutrition security to 2050, depending on the IMPACT model, yet focusing on food prices, per capita kilocalorie demand, the level of hunger, and micronutrient availability. The noted information is the reference scenario that considers various changes in population, income, and climate to make the case that there will be significant regional output advantages in the alleviation of hunger. Nevertheless, the growth is lower compared to the SDG 2.1 objective of halting hunger in the year 2030. The main processes that have been leading to increased food security include the growing population, increasing GDP, crop yield, and global warming. Comparison of sensitivity analysis of alternative scenarios revealed that the GDP and population change most significantly affect food security as compared to the rate of climate change and the other climate models (GCMs). It is estimated that the CO_2_ fertilization can reduce the number of starving people by 14 percent by the year 2050 compared to the situation that would arise in the case of a zero CO_2_ fertilization setting. The analysis shows that there is a need to make additional policies and investments to combat hunger. Agricultural research and development can be enhanced to increase the productivity of crops at cheap prices in a bid to enhance food security. Concerning demand, the adoption of sustainable diets full of Micronutrients and the reduction in the take-up of high-energy foods have remained a concern. Besides economic growth and production, specialized agricultural policies and social security nets are also required to achieve food security and adequate nutrition. Notwithstanding the challenges these transformations have caused, they are becoming highly pressing and most importantly in low- and middle-income countries where the poor and vulnerable communities face the worst issues. To address these challenges, it is important to address major questions to create a balance between goals and solutions to underline the importance of cooperation to the development of a more sustainable and fair food future.

## Data Availability

The raw data supporting the conclusions of this article will be made available by the authors, without undue reservation.
